# Enhanced and cross-reactive *in vitro* memory B cell response against Epstein-Barr virus nuclear antigen 1 in multiple sclerosis

**DOI:** 10.3389/fimmu.2024.1334720

**Published:** 2024-08-27

**Authors:** Zoe Marti, Josefine Ruder, Olivia G. Thomas, Mattias Bronge, Lorenzo De La Parra Soto, Hans Grönlund, Tomas Olsson, Roland Martin

**Affiliations:** ^1^ Institute of Experimental Immunology, University of Zurich, Zurich, Switzerland; ^2^ Research and Development, Cellerys, Schlieren, Switzerland; ^3^ Department of Neuroimmunology and Multiple Sclerosis Research, University Hospital Zurich, Zurich, Switzerland; ^4^ Therapeutic Immune Design Unit, Department of Clinical Neuroscience, Karolinska Institutet, Stockholm, Sweden; ^5^ Department of Neurology, Karolinska University Hospital, Stockholm, Sweden; ^6^ Department of Medical Biochemistry and Microbiology, Uppsala University, Uppsala, Sweden; ^7^ Neuroimmunology Unit, Department of Clinical Neurocience, Karolinska Institutet, Stockholm, Sweden

**Keywords:** multiple sclerosis, molecular mimicry, memory B cells, Epstein-Barr virus, EBNA1, anoctamin-2, natalizumab, GlialCAM

## Abstract

Multiple sclerosis (MS) is a prototypical autoimmune disease of the central nervous system (CNS). In addition to CD4^+^ T cells, memory B cells are now recognized as a critical cell type in the disease. This is underlined by the fact that the best-characterized environmental risk factor for MS is the Epstein-Barr virus (EBV), which can infect and persist in memory B cells throughout life. Several studies have identified changes in anti-EBV immunity in patients with MS. Examples include elevated titers of anti-EBV nuclear antigen 1 (EBNA1) antibodies, interactions of these with the MS-associated HLA-DR15 haplotype, and molecular mimicry with MS autoantigens like myelin basic protein (MBP), anoctamin-2 (ANO2), glial cell adhesion molecule (GlialCAM), and alpha-crystallin B (CRYAB). In this study, we employ a simple *in vitro* assay to examine the memory B cell antibody repertoire in MS patients and healthy controls. We replicate previous serological data from MS patients demonstrating an increased secretion of anti-EBNA1_380-641_ IgG in cell culture supernatants, as well as a positive correlation of these levels with autoantibodies against GlialCAM_262-416_ and ANO2_1-275_. For EBNA1_380-641_ and ANO2_1-275_, we provide additional evidence suggesting antibody cross-reactivity between the two targets. Further, we show that two efficacious MS treatments – natalizumab (NAT) and autologous hematopoietic stem cell transplantation (aHSCT) – are associated with distinct changes in the EBNA1-directed B cell response and that these alterations can be attributed to the unique mechanisms of action of these therapies. Using an *in vitro* system, our study confirms MS-associated changes in the anti-EBNA1 memory B cell response, EBNA1_380-641_ antibody cross-reactivity with ANO2_1-275,_ and reveals treatment-associated changes in the immunoglobulin repertoire in MS.

## 1 Introduction

Multiple Sclerosis (MS) is a prototypic organ-specific autoimmune disease of the central nervous system (CNS) (1) that mainly manifests in young individuals and particularly in women (2). The etiology comprises a complex interaction between genetic risks, particularly the HLA-DR15 haplotype (3, 4), and several environmental risk factors including infection with the Epstein-Barr virus (EBV) (5–7), low vitamin D, smoking, and adolescent obesity. Protective factors include HLA-A*0201 or infection with cytomegalovirus (CMV) (2). In the past years, EBV infection has received strong interest in the field of MS research due to a recent study, which presented compelling evidence that transition to an EBV-seropositive status precedes MS onset (8). This observation highlighted the virus’ role in causing the disease. CD4^+^ T cells were long considered the most important pathogenic cellular compartment in MS (1). However, the high efficacy of B cell-depleting therapies together with mechanistic studies have now firmly established B cells – primarily memory (CD27^+^) B cells – as a key cell type in MS (9–12). Hence, both B and T lymphocytes are important in the disease pathogenesis and most likely act in a cooperative manner. In line with this, lymphocyte migration across the blood-brain barrier (BBB) forms an essential step in MS onset and relapses (13, 14). The importance of this event is highlighted by the efficacy of the MS treatment natalizumab (NAT), a humanized monoclonal antibody that prevents the adhesion and extravasation of activated lymphocytes across the BBB (15). Multiple other effective treatments have been approved for MS. Important examples include monoclonal antibodies against CD20 (16) which deplete B cells and indirectly also affect CD4^+^ T cells, or against CD52, which depletes both B- and T cells (17). Another approach called autologous hematopoietic stem cell transplantation (aHSCT) eradicates the entire immune system followed by the reconstitution of a tolerant B- and T cell repertoire (18, 19). aHSCT not only blocks disease activity for long periods and in a large percentage of individuals (18) but may even present a cure to the disease at least in some patients.

Although the contribution of memory B cells in MS is not fully resolved yet, multiple mechanisms of action appear to be involved. The most important examples include the secretion of proinflammatory cytokines (20, 21), migration to the CNS (10, 22), the formation of tertiary lymphoid follicle-like structures in the meninges (23), and the presentation of (auto)antigens to CD4^+^ T cells as antigen-presenting cells (APCs) (11, 24, 25). As APCs, B cells are involved in the activation, proinflammatory differentiation, expansion, and migration of autoreactive CD4^+^ T cells into the CNS (11). Since EBV-infected B cells express virus-derived antigens (26), EBV epitopes may present important immunogenic targets in this lymphocyte cooperation (27). Furthermore, EBV-directed immune responses may also target host autoantigens by a mechanism referred to as molecular mimicry, i.e. the cross-recognition of a foreign and an autoantigen by a T or B cell receptor/antibody (28). In MS, there is growing evidence that EBV proteins, and particularly the EBV nuclear antigen 1 (EBNA1), are involved in cross-reactivity with multiple MS autoantigens. Cross-reactivity for EBNA1 has been demonstrated with myelin basic protein (MBP) (29–31), anoctamin-2 (ANO2) (32), glial cell adhesion molecule (GlialCAM) (33), and alpha-crystallin B (CRYAB) (34), both for B cell antibodies and/or CD4^+^ T cells.

In this study, we adopted a previously described *in vitro* system (35) to characterize the memory B cell phenotype, antibody repertoire, and specificity in untreated and NAT-treated relapsing-remitting MS (RRMS) patients, MS patients undergoing aHSCT, as well as healthy donors (HDs). We set our primary focus on identifying EBNA1-associated patterns in the memory B cell repertoire, including quantitative changes in EBNA1 antibodies as well as their cross-reactivity with the MS autoantigens ANO2, CRYAB, and GlialCAM. We further assessed how two highly effective MS treatments with different modes of action, i.e. NAT and aHSCT, differentially affect the immunoglobulin repertoire directed against EBNA1. The results from our *in vitro* experiments confirm previous reports of an elevated secretion of anti-EBNA1 antibodies in MS patients (36, 37) and corroborate the cross-reactivity between the C-terminal end of this protein (aa 380-641) with MS-associated autoantigens. Our findings not only demonstrate that the employed *in vitro* system is suitable for studying disease-associated patterns in the antibody repertoire, but they further imply that EBV immunity is directly affected by different treatment interventions.

## 2 Materials and methods

### 2.1 Patient samples

Samples were collected from untreated RRMS patients (RRMS), NAT-treated RRMS patients (NAT), MS patients undergoing aHSCT (aHSCT), as well as healthy donors (HD) (**Supplementary Table 1**). Peripheral blood samples, serum, and leukaphereses were obtained with informed consent under ethics protocols EC-No. 2013-0001, EC-No. 2014-0699, and patients receiving aHSCT under protocol BASEC-No. 2018-01854 by the Cantonal Ethics Committee of Zurich, Switzerland. Five samples (RRMS17, NAT1, NAT9, HD15, HD18) were excluded from analyses due to insufficient lymphocyte viability (≤60%) as determined by flow cytometry analysis after stimulation with IL-2 and R848 as outlined in the methods section “Memory B Cell Activation and Expansion”. The final group sizes were n = 16 for HDs, n = 19 for RRMS patients, n = 10 for NAT patients, and n = 15 for aHSCT patients. Age and sex were nearly balanced across cohorts (**Supplementary Table 1**). Peripheral blood mononuclear cells (PBMCs) were isolated according to standard laboratory procedures (38) from blood, buffy coat, or leukapheresis using density centrifugation. Isolated PBMCs were cryopreserved in freezing medium (heat-inactivated FCS + 10% DMSO) at -80°C for 24h to 48h and subsequently stored in liquid nitrogen (-180°C) until use. Serum samples were centrifuged for 13min at 1700g, aliquoted, and stored at -80°C.

### 2.2 Assessment of EBV viral load and reactivation

The presence or absence of EBV reactivation after aHSCT was assessed in EDTA-anticoagulated whole blood samples at the Department of Clinical Virology, University of Zurich, using clinical routine quantitative PCR (qPCR) for the detection of EBV DNA. DNA was extracted from whole blood using NucliSENS (BioMérieux, Marcy-l’Étoile, France) according to the manufacturer’s instructions. Quantification of EBV DNA copies was performed by TaqMan real-time PCR technique (Applied Biosystems, Massachusetts, USA) as described previously (39) with modified primers for the conserved BamH1 W fragment of EBV (5′-CTTCTCAGTCCAGCGCGTTT-3′ and 5′-CAGTGGTCCCCCTCCCTAGA-3′) and a fluorogenic probe (5′-FAM CGTAAGCCAGACAGCAGCCAATTGTCAG-TAMRA-3′). The PCR was run on an ABI Prism 7700 Sequence Detector (Applied Biosystems, Massachusetts, USA), and samples were analyzed in duplicates. The lower limit of quantification was 122 copies/ml of EBV DNA. Samples with <122 copies/ml of EBV DNA were considered negative for EBV DNA. For 7 out of 8 aHSCT patients with a reactivation (≥122 copies/ml), the confirmation occurred within 3 months after aHSCT. Only in one case (aHSCT25) was it confirmed after 1.5 years. No case of EBV reactivation was associated with a symptomatic course.

### 2.3 Memory B cell activation and expansion

#### 2.3.1 Cell culture seeding (day 0)

Cryopreserved PBMCs were thawed in complete RPMI medium (cRPMI; RPMI + 1% L-glutamine + 1% Hepes + 1% Penicillin-Streptomycin + 0.1% Gentamicin + 10% FCS), spun down for 5min at 300g, resuspended in phosphate-buffered saline (PBS) and counted. 1x10^6^ of resuspended cells were withdrawn and stored at 4-8°C for baseline flow cytometry analysis at day 0. After another centrifugation step (as above), cells were resuspended in cRPMI to a final concentration of 1x10^6^ cells/ml. Cells from each donor were split into two fractions, one for a verum (V) and one for a non-stimulated control condition (C). For the selective activation and expansion of memory B cells and their differentiation into antibody-secreting cells (ASCs), the verum fraction of PBMCs were supplemented with 1’000 U/ml IL-2 (Proleukin^®^, Yardley, USA) and 2.5μg/ml R848 (Invivogen, Toulouse, France) as described by Pinna et al. (35). Other than in the original protocol, we used 24-well flat-bottom plates and a higher seeding density of 1x10^6^ PBMCs/ml. Non-stimulated controls were seeded in cRPMI without IL-2 or R848. Both conditions were at least plated in duplicates. Cell cultures were incubated at 37°C, 5% CO_2_ for one week (day 0 – day 7). In addition, cells were frequently examined for morphological changes under an inverted light microscope.

#### 2.3.2 Cell and supernatant harvest (day 7)

On day 7, the cell culture supernatants from all samples were carefully aspirated. Replicates were pooled and then stored at -20°C. Cells were harvested by repeatedly flushing each well with cRPMI and pooling the content of replicate wells. Suspensions were spun down for 5min at 300g, resuspended in PBS, and counted. For each sample and condition, 1x10^6^ cells were removed and kept at 4-8°C until flow cytometry analysis. If fewer viable cells were available, the entire sample was used for flow cytometry staining.

### 2.4 Enzyme-linked immunosorbent assays at the University Hospital of Zurich, Switzerland

Several commercially available ELISAs were performed at the University Hospital of Zurich, Switzerland. These included kits for the quantification of total IgG, total IgM, as well as antigen-specific IgG against EBNA1, the EBV viral capsid antigen (VCA), and tetanus toxoid (TT) (**Table 1**). For quantification of IgM and IgG, plates were pre-coated according to the manufacturer’s protocol using 96-well flat bottom plates (Costar Polystyrene 96-well assay plate, CORNING). For antigen-specific assays, pre-coated plates provided by the respective kit were used. Assays were performed following the manufacturer’s protocol. Samples were plated in duplicates, including blanks and standards, which were included on every individual plate. Optical density (OD) was measured using the Synergy H1 microplate reader and the Gen5 Software (BioTek), both at the individual target wavelength (OD_target_) specified by the manufacturer, and a reference wavelength of 650nm (OD_ref_). For data analysis, reference ODs and the average OD values from blanks (OD_blank_) were subtracted from each reading to obtain adjusted ODs (OD_adj_ = OD_target_ – OD_ref_ – OD_blank_). If adjusted OD values were negative, values were set to zero. Sample types, dilutions, and details on absorbance measurements are summarized in **Table 1**.

**Table 1:** Summary of materials and parameter settings for in-house ELISAs at the University Hospital of Zurich, Switzerland.

ELISA kit	Provider	Cat. Nr.	Sample type	Sample Dilution	WL_T_ [nm]
**Human IgG ELISA^basic^ Kit**	Mabtech, Nacka Strand, Sweden	3850-1AD-6	SN+/- Serum	SN+: 1:400-1:1600 SN-: Up to 1:5 Serum: 1:10^5^-1:5x10^5^	405
**Human IgM ELISA^basic^ Kit**	Mabtech, Nacka Strand, Sweden	3880-1AD-6	SN+/-	SN+: 1:400-1:600 SN-: Up to 1:5	405
**Abnova™ Epstein Barr Virus EBNA-1 IgG ELISA Kit**	Abnova, Taipei, Taiwan	KA1448	SN+/- Serum	SN+: Undiluted Serum: 1:200-1:400	450
**Abnova™ Epstein Barr Virus VCA IGG ELISA Kit**	Abnova, Taipei, Taiwan	KA1444	SN+/-	SN+: Undiluted SN-: Undiluted	450
**Human Tetanus Toxoid Antibody IgG (TT-IgG) ELISA Kit**	MyBioSource, San Diego, USA	9907877	SN+/-	SN+: Undiluted SN-: Undiluted	450

SN+, Cell culture supernatants after stimulation *in vitro*; SN-, Cell culture supernatants without stimulation *in vitro*; WL_T_, Target wavelength specified by the manufacturer.

### 2.5 External ELISAs and antibody blocking assays at the Karolinska Institute, Sweden

The previously outlined ELISA experiments were independently replicated at the Center for Molecular Medicine of the Karolinska Institute (Stockholm, Sweden) using a custom ELISA with recombinant *E. coli* in-house-produced proteins. EBNA1 aa1-120, EBNA1 aa380-641, ANO2 aa1-275, and GlialCAM aa262-416 were produced and purified as previously described (34). ELISA half-well plates (Costar Polystyrene 96-well assay plate, CORNING) were coated with the antigens or bovine serum albumin (BSA, HyClone, Cytivia) at 2.5µg/ml at 4°C overnight before blocking using PBS with 1% w/v BSA and 0.05% Tween-20 for 1h at room temperature. After washing, cell culture supernatants (harvested after stimulation with IL-2 and R848 as described above) were diluted 1:5 in ELISA buffer (PBS + 0.2% w/v BSA + 0.05% Tween-20), added to the plates, and incubated for 2h at room temperature. The plates were washed and a secondary HRP-conjugated anti-IgG antibody (Goat anti-human IgG, HRP conjugated, 62-8420, Invitrogen) diluted 1:2000 in ELISA buffer was added for 1h at room temperature in the dark. Following incubation, the plates were washed and TMB-substrate (TMB Super Slow, T5569, Sigma-Aldrich) was added for 15min before the reaction was stopped with an equal volume of 0.5M H_2_SO_4_. For the blocking experiments, supernatants were incubated with 30μM of EBNA1 aa380-641 protein or PBST (PBS + 0.05% Tween™ 20) overnight at 4°C. The following day, blocked samples were diluted 1:5 in ELISA buffer and developed as above. The OD was read at 450 nm using a SpectraMax Plus 384 (Molecular Devices, San Jose, USA) with the SoftMax Pro 7.0.3 software (Molecular Devices, San Jose, USA). All tests were run in duplicates with background (BSA) in quadruplicates. Before analysis, individual background ODs (BSA) were subtracted from antigen OD, and if the resulting values were negative, they were set to zero (= OD_adj_). Supernatants were run sequentially, and the three different cohorts were proportionally distributed on each assay plate.

### 2.6 Flow cytometry

Flow cytometry was performed on days 0 and 7 of the PBMC expansion protocol using the markers summarized in **Table 2**. For the staining of cells on days 0 and 7, up to 1x10^6^ cells were suspended in PBS and spun down for 5min at 300g. The pellet was resuspended in 100μl of a live/dead staining mix consisting of 0.1% LIVE/DEAD™ Fixable Near-IR Dead Cell Stain (Thermo Fisher Scientific, Eugene, OR, USA, Cat. Nr. 10119), 20% purified human IgG for blocking (Sigma Aldrich, St. Louis, MO, USA, Cat. Nr. I4506) and PBS. Cells were incubated for 30min at 4-8°C, washed with 2ml FACS buffer (PBS + 1% FCS + 0.4% EDTA), and spun down as above. After discarding the supernatant, cells were resuspended in a total volume of 100μl of antibody staining mix containing all fluorophore-antibody conjugates (**Table 2**) in FACS buffer. The tubes were incubated and protected from light at room temperature for 20min. After an additional washing and centrifugation step as above, stained cells were ultimately resuspended in 300μl FACS buffer and kept on ice until flow cytometry. Readouts were acquired using the LSR FortessaTM Flow Cytometer (BD Biosciences). Gating was performed using FlowJo™ Software. General gating included the selection of lymphocytes according to size (FSC-A) and granularity (SSC-A), live cells, and singlets (FSC-A vs. FSC-H). Subsequently, marker-specific gates were set as outlined in **Supplementary Figure 1**. In addition to gating on various CD19^+^ B cell subsets, we accounted for ungated CD19^+^ events by subtracting the total frequency of B cells within lymphocytes from the sum of all gated CD19^+^ events. We referred to this “ungated” CD19^+^ population as “other B cells”. Lymphocyte viability was quantified according to the live/dead staining. As a quality control, only samples with a viability of >60% after stimulation *in vitro* were included in further analyses (**Supplementary Figure 2**).

**Table 2:** Summary of antibody-fluorophore conjugates and live/dead staining material used for flow cytometry analysis in the *in vitro* experiments.

Marker	Fluorophore	Isotype	Clone	Dilution	Provider	Catalog number
**CD3**	AF700	Mouse IgG1, κ	HIT3a	1/100	Biolegend	300324
**IgD**	PE	Mouse IgG2a, κ	IA6-2	1/10	BD Biosciences	555779
**IgM**	FITC	Mouse IgG1, κ	MHM-88	1/40	Biolegend	314506
**CD27**	HorPE CF594	Mouse IgG1, κ	M-T271	1/50	BD Biosciences	562297
**CD24**	BV421	Mouse IgG2a, κ	ML5	1/20	Biolegend	311121/2
**CD38**	BV711	Mouse IgG1, κ	HIT2	1/100	Biolegend	303527/8
**CD138**	APC	Mouse IgG1, κ	MI15	1/10	Biolegend	356505/6
**HLA-DR**	PE-Cy7	Mouse IgG2a, κ	L243	1/200	Biolegend	307615/6
**CD11c**	BV785	Mouse IgG1, κ	3.9	1/400	Biolegend	301643/4
**CD19**	PerCP-Cy5.5	Mouse IgG2a, κ	HIB19	1/25	Biolegend	302229/30
**L/D**	(near IR)	-	-	1/1000	Invitrogen	L10119

L/D, Live/Dead staining; IR, infrared.

### 2.7 Statistical analyses

Cell frequencies and counts from flow cytometry analyses and ODs obtained from ELISAs were both preliminarily examined in Microsoft^®^ Excel (Version 16.61.1). ELISA standard curves and conversion of OD values to quantitative units were also performed using this software. All calibration curves produced a R^2^ > 0.99 and were generated using a 2- or 3-parameter polynomic fit. For statistical analyses, data was analyzed using RStudio software (Version 2023.12 + 369, Posit Software, PBC). Comparisons between cohorts were evaluated using Wilcoxon rank-sum tests (Mann-Whitney-U tests), and Wilcoxon signed-rank tests were used for paired within-subject readouts. Multiple pairwise comparisons were adjusted using the Holm’s method. Linear regressions were calculated using Spearman’s rank correlation. *P values* were considered significant at ≤ 0.05. Further significance levels were defined as ** p ≤ 0.01, *** p ≤ 0.001, and **** p ≤ 0.0001. Non-significant results were labelled as “ns”.

## 3 Results

### 3.1 IL-2 and R848 selectively expand and activate memory-derived antibody-secreting B cells

**Figure 1:**
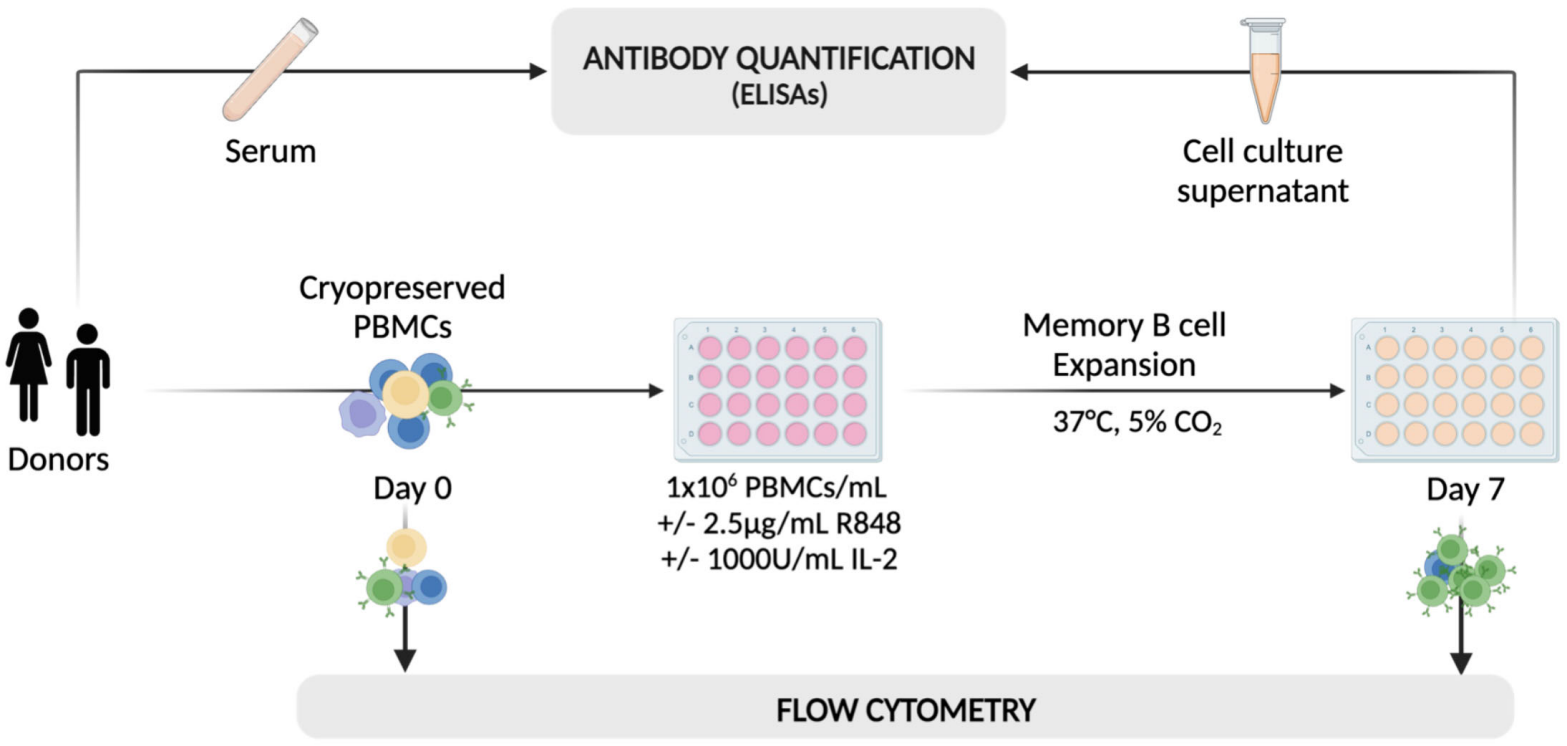
A schematic of the workflow adopted for the selective activation and expansion of CD27^+^ memory B cells using 2.5μg/ml of R848 and 1’000 U/ml of IL-2. On day 0, cryopreserved PBMCs from HDs and MS patients were analyzed using flow cytometry and plated at 10^6^ cells/well with (+) or without (–) the addition of IL-2 and R848. After one week, cells from stimulated (Day 7 +) and non-stimulated wells (Day 7 -) were analyzed using the same flow cytometry protocol as on day 0. On the same day, cell culture supernatants were collected for analysis using a range of immunosorbent assays. Serum samples from some patients were also included in the analysis to assess the comparability between antibody titers in serum and cell culture supernatants obtained from the *in vitro* expansion. Created with BioRender.com.

**Figure 2:**
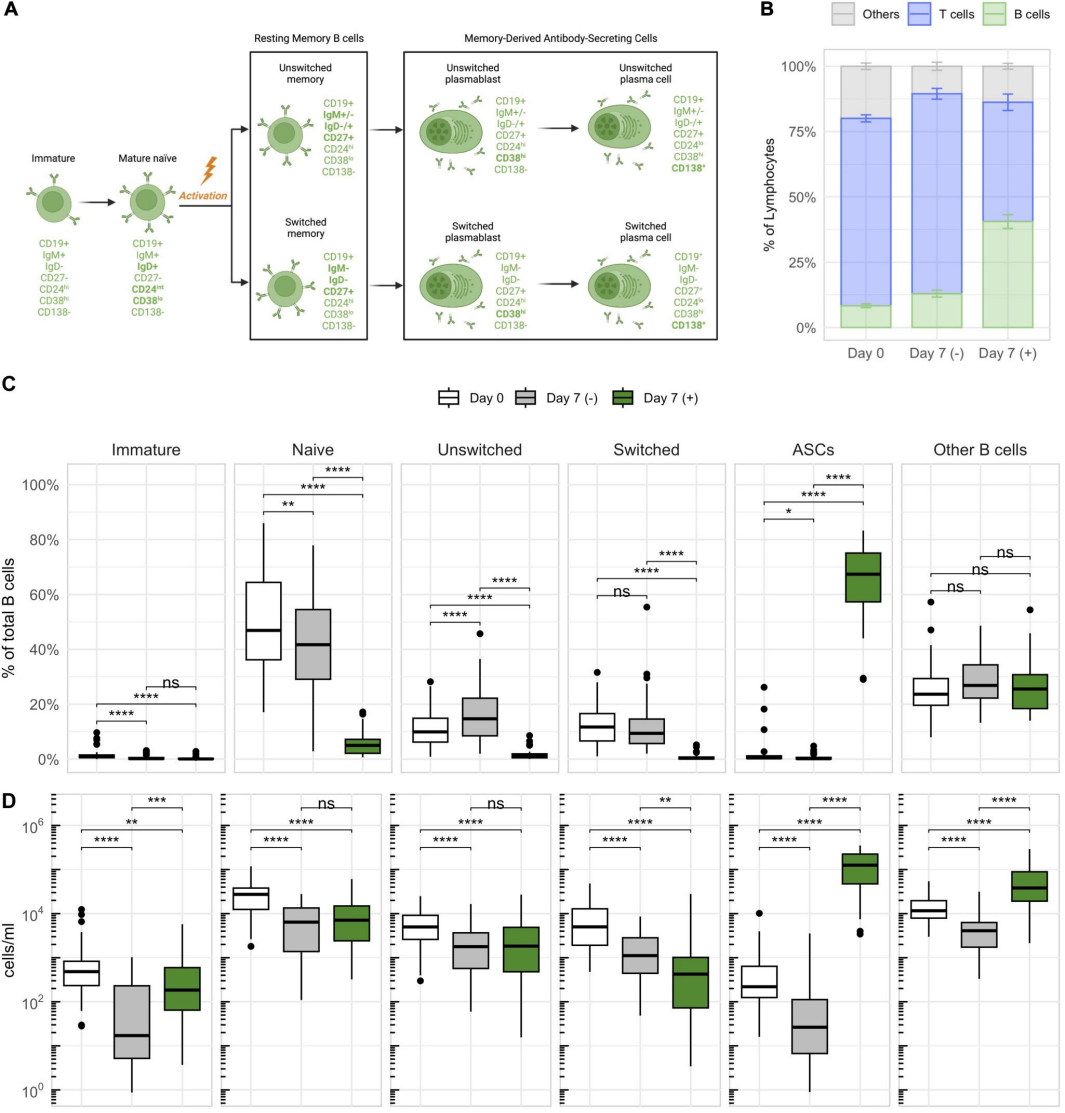
Stimulation with IL-2 and R848 stimulates the expansion of CD27^+^ antibody-secreting B cells (ASCs) within PBMCs. **(A)** Characterization and nomenclature of B cell differentiation stages according to the expression of distinct surface markers. Key changes distinguishing a subset from its previous differentiation stage are highlighted in bold. The illustration was created with *BioRender.com*. **(B)** The average frequency of major lymphocyte compartments with standard errors across all donors on day 0 and on day 7 with (+) or without (–) stimulation. The subset referred to as “Others” was calculated by subtracting the percentages of B and T cells within lymphocytes from 100%. **(C)** The relative frequencies (as percentages of total B cells) and **(D)** concentrations of different B cell subsets based on the differentiation scheme outlined in **(A)**. p = Wilcoxon signed rank tests with Holm’s correction, n = 45. * p ≤ 0.05,** p ≤ 0.01, *** p ≤ 0.001, and **** p ≤ 0.0001. P-values above 0.05 were considered non-significant (ns).

With the aim to examine the differentiation and function of B cell populations, particularly memory B cells and antibody-secreting B cells (ASCs) in patients with MS *in vitro*, we adopted a protocol published by Pinna et al. (35). This comprised a dual stimulation with a toll-like receptor (TLR) 7/8 agonist (R848) and interleukin-2 (IL-2) for the selective expansion and activation of CD27^+^ memory B cells (**Figure 1**). In short, we expanded whole peripheral blood mononuclear cells (PBMCs) from untreated relapsing-remitting MS (RRMS) patients (n_RRMS_ = 19), NAT-treated RRMS patients (n_NAT_ = 10), and healthy donors (n_HD_ = 16) by addition of R848 at 2.5µg/ml and IL-2 at 1’000 IU/ml. On day 7, we assessed cellular phenotypes by flow cytometry, focusing on various B cell differentiation stages (**Figure 2A**). In addition, cell culture supernatants were harvested on day 7 for immunosorbent assays. Five samples were excluded from further analyses due to poor cell viability (≤60% of live lymphocytes) after stimulation (**Supplementary Figure 2**).

Stimulated cultures showed the expansion of B cells, which occurred at the expense of other lymphocytes such as T cells (**Figure 2B**). Within B cells, memory-derived (CD27^+^) ASCs expanded the most, accounting for 65 ± 13.1% of B cells after stimulation (**Figures 2C, D**). These expanded ASCs consisted almost exclusively of short-lived plasmablasts (CD138^–^), and only a minor fraction of long-lived plasma cells (CD138^+^) (**Supplementary Figure 3A**). Additionally, they were predominantly class-switched (IgD^–^IgM^–^) (**Supplementary Figure 3B**). CD3^+^ T cells showed upregulation of surface HLA-DR, most notably in NAT patients, and a significant reduction of CD27^+^ T cells in response to stimulation (**Supplementary Figure 4**). These changes indicated that T cells also respond to the stimulation, despite not being expanded. In conclusion, the dual stimulation with IL-2 and R848 efficiently expanded B cells, and primarily memory-derived CD27^+^ ASCs, within total PBMCs. The assay was therefore well suited for a more detailed investigation of memory B cells.

### 3.2 Switched memory B cells are more abundant in MS patients compared to healthy controls

**Figure 3:**
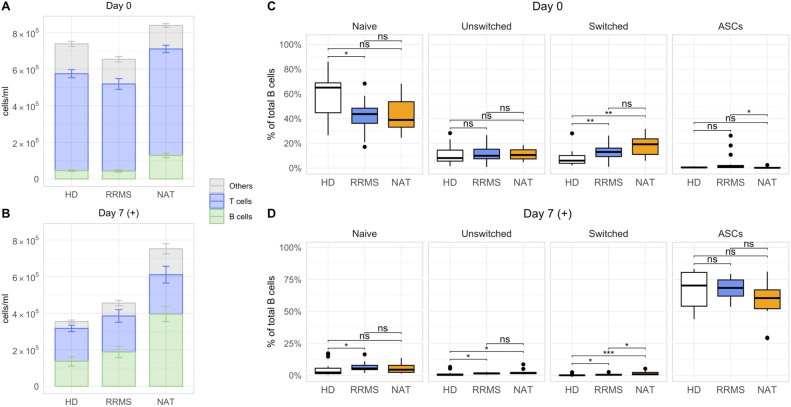
Differences in the composition of lymphocytes and B cells between HDs, untreated RRMS patients and NAT patients. **(A, B)** The mean lymphocyte numbers with standard errors on day 0 **(A)**, and after stimulation on day 7 **(B)**. The subset referred to as “Others” was calculated by subtracting the concentration of CD19^+^ B and CD3^+^ T cells from the total lymphocyte concentration. **(C, D)** The relative frequencies of different B cell subsets before **(C)** and after stimulation with R848 and IL-2 *in vitro* **(D)**. p = Wilcoxon rank-sum tests with Holm’s correction. * p ≤ 0.05,** p ≤ 0.01, *** p ≤ 0.001. P-values above 0.05 were considered non-significant (ns).

After confirming that the stimulation with IL-2 and R848 successfully expanded CD27^+^ ASCs within PBMCs, we focused on identifying phenotypic and stimulation-dependent variations among the cohorts (**Figure 3**). Consistent with prior data (40), B cells were overall most abundant in NAT patients, both in untreated PBMCs before stimulation (**Figure 3A**) as well as after stimulation (**Figure 3B**). Due to the higher numbers of B cells in this group, we focused on a comparison of the relative frequencies of B cell subsets. In untreated samples prior to stimulation, MS patients showed an increased abundance of switched memory B cells and a slightly lower percentage of naïve B cells compared to HDs (**Figure 3C**). After stimulation, the median frequencies of both naïve and resting memory B cells (unswitched and switched) dropped below a median of 2% across all cohorts, which paralleled the stimulation-induced appearance of ASCs (**Figure 3D**). Despite some statistically significant differences between cohorts, the low frequencies indicated minimal relevance of the respective subsets. There was no difference in the frequency of expanding ASCs after stimulation between the three groups. In conclusion, samples from both untreated and NAT-treated RRMS patients presented with lower frequencies of naïve, and higher frequencies of switched memory B cells compared to HDs. The stimulation with IL-2 and R848 led to a consistent and significant expansion of ASCs in all cohorts.

### 3.3 Stimulated B cells secrete high amounts of IgG and IgM *in vitro*

**Figure 4:**
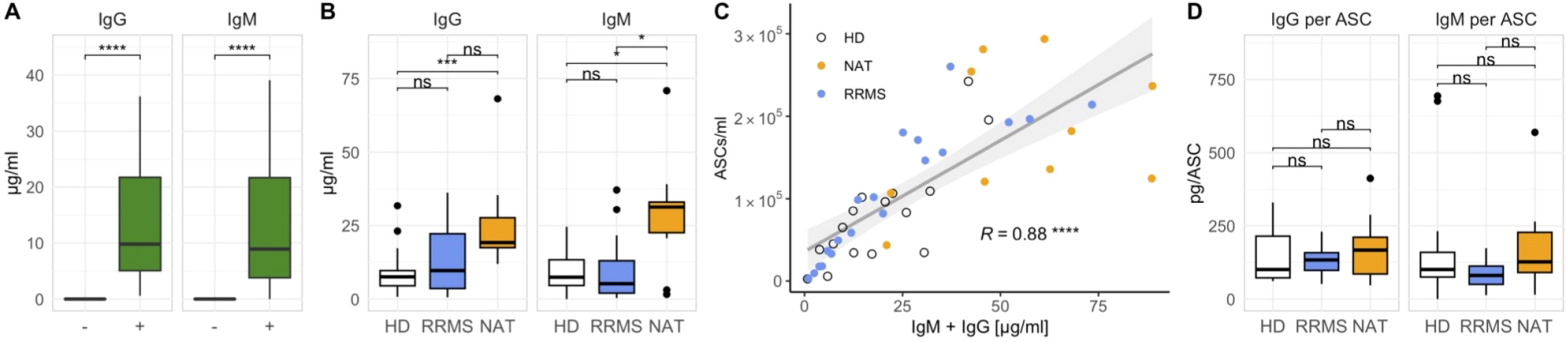
The secretion of IgM and IgG increases after stimulation with IL-2 and R848 and correlates with the number of ASCs. **(A)** IgM and IgG quantities in stimulated (+) and unstimulated (–) cell cultures on day 7 across all donors (p = Wilcoxon signed-rank tests). **(B)** Differences in IgM and IgG concentrations between cohorts after stimulation (p = Wilcoxon rank-sum tests with Holm’s correction). **(C)** Linear regression analysis comparing the cumulative production of IgG and IgM with the number of ASCs (p = Spearman’s rank correlation). **(D)** Immunoglobulin secretion per ASC after stimulation (p = Wilcoxon rank-sum tests with Holm’s correction). * p ≤ 0.05,** p ≤ 0.01, and **** p ≤ 0.0001. P-values above 0.05 were considered non-significant (ns).

Next, we assessed the antibody secretion *in vitro*. Supernatants from stimulated cell cultures contained significantly higher amounts of both total IgM and IgG compared to non-stimulated controls (**Figure 4A**). NAT-treated patients produced significantly more immunoglobulins compared to both other groups (**Figure 4B**), which coincided with their higher absolute ASC counts (**Supplementary Figure 5**, far right panel). ASC numbers correlated well with the cumulative amount of IgM + IgG in the supernatants (**Figure 4C**), and despite the variation in antibody secretion per ml, there was no significant difference in the secretion of either IgG or IgM per ASC between the three groups (**Figure 4D**). Hence, we confirmed that the addition of IL-2 and R848 *in vitro* not only expanded CD27^+^ ASCs but also resulted in their functional activation.

### 3.4 Stimulated B cells from MS patients produce more IgG against EBNA1_380-641_ and MS autoantigens compared to healthy donors

**Figure 5:**
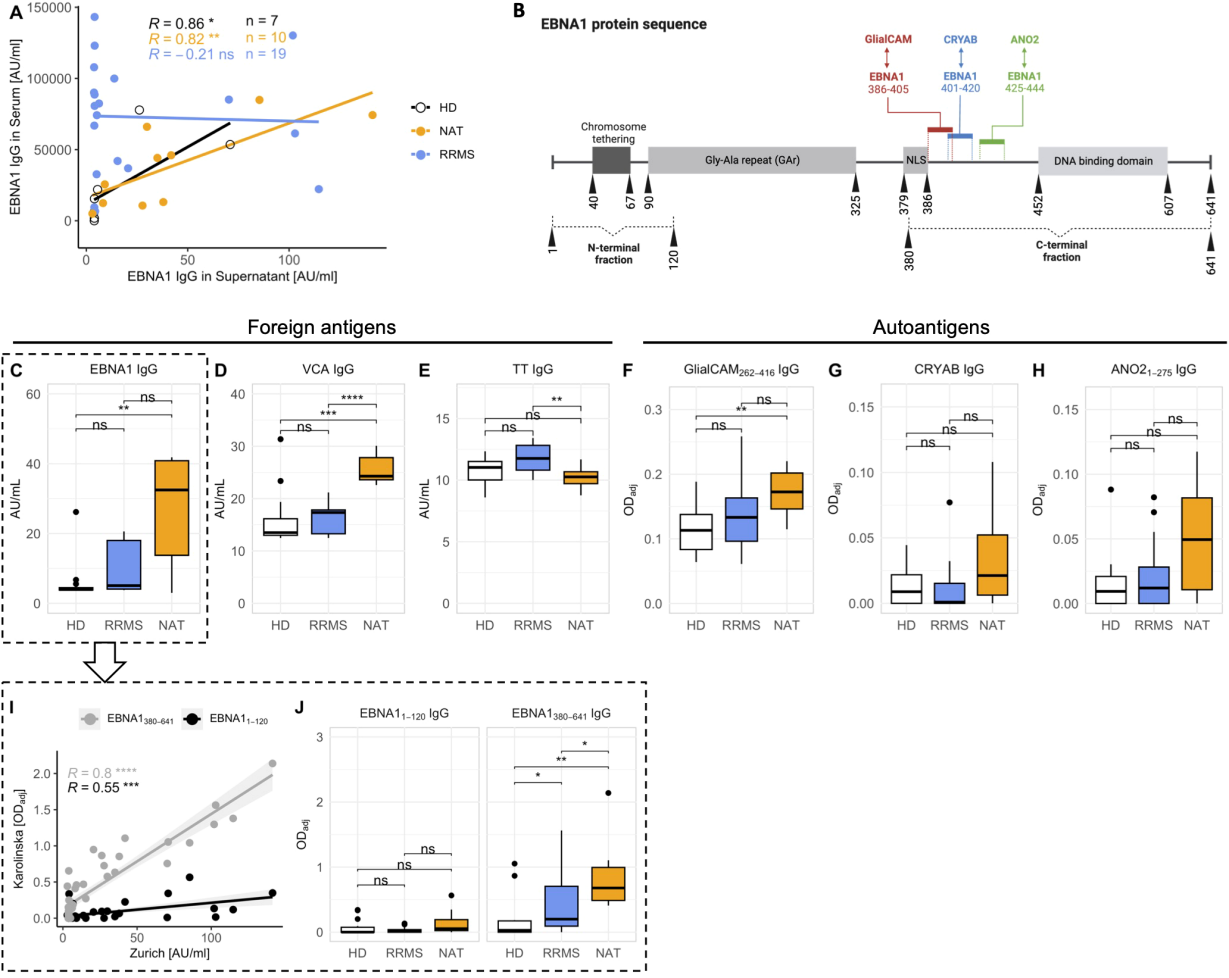
*In vitro* secretion of antibodies against foreign antigens and MS autoantigens. **(A)** Correlation between anti-EBNA1 IgG levels in serum and in supernatants after stimulation *in vitro* (p = Spearman’s rank correlation). Note that sera from HDs were only available from a limited number of individuals (n = 7 out of 16). **(B)** Schematic of the EBNA1 amino acid sequence. Details were obtained from UniProt entry EBNA1_EBVB9 (P03211) Colored bars mark the EBNA1 amino sequences for which antibody cross-reactivity was shown previously (refer to text for literature citations). Vertical numbers indicate amino acid positions. For a more detailed dissection of the antibody response against EBNA1, humoral responses against the N- (aa1-120) and C-terminal (aa380-641) fraction of the protein were assessed separately (subfigures **I, J**). The illustration was created with *BioRender.com*. **(C-H)** Levels of antigen-specific IgG levels in cell culture supernatants after stimulation *in vitro*. **(I)** Linear regression analysis comparing the levels of total anti-EBNA1 IgG with IgG levels against the N- and C-terminal region of the antigen (p = Spearman’s rank correlation). **(J)** Comparison of the antibody quantities targeting the N- and C-terminal ends of EBNA1 in the three cohorts (p = Wilcoxon rank-sum tests with Holm’s correction). NLS, Nuclear Localization Signal. * p ≤ 0.05,** p ≤ 0.01, *** p ≤ 0.001, and **** p ≤ 0.0001. P-values above 0.05 were considered non-significant (ns).

To obtain a better understanding of the *in vitro* antibody response after stimulation, we examined antigen-specific IgG secretion with a focus on targets showing potential cross-reactivity in MS. Based on accumulating data suggesting molecular mimicry between the EBV antigen EBNA1 and several MS-autoantigens for both CD4^+^ T cells (29, 34) as well as antibodies (32–34), we focused on EBNA1. We quantified anti-EBNA1 IgG in both stimulated cell culture supernatants as well as in sera of the same patients using immunosorbent assays. The levels of anti-EBNA1 IgG in supernatants and sera correlated significantly in the case of HDs and NAT patients but not in untreated RRMS patients (**Figure 5A**). In addition to EBNA1, we quantified the *in vitro* IgG secretion against two other foreign antigens, including the EBV viral capsid antigen (VCA) and the common vaccine antigen tetanus toxoid (TT), which served as an EBV-independent control (**Figures 5C–E**). Regarding autoantigens, we selected three targets for which cross-reactivity with EBNA1 antibodies had been described previously. These included GlialCAM aa262-416 (33), CRYAB (34) and ANO2 aa1-275 (32) (**Figures 5F–H**), all of which appear to cross-react with specific EBNA1 epitopes situated C-terminal of the Glycine-Alanine Repeat (GAr) domain of the protein (**Figure 5B**).

IgG levels against both EBV antigens – EBNA1 and VCA – were markedly elevated in NAT-treated patients (**Figures 5C, D**). Interestingly, anti-TT IgG levels were lowest in this group (**Figure 5E**). As expected, the levels of antibodies against autoantigens were generally low (**Figures 5F–H**) but all three were highest in NAT-treated patients, and even significantly different from HDs in the case of anti-GlialCAM IgG (**Figure 5F**). However, these differences were much less pronounced after normalizing the individual antigen-specific IgG levels to total IgG (**Supplementary Figure 6**).

For further characterization of the *in vitro* anti-EBNA1 IgG responses, we next quantitated IgG against EBNA1 fragments located N- (aa1-120) or C-terminal (aa380-641) of the GAr domain, respectively (**Figure 5B**). The levels of total anti-EBNA1 IgG (**Figure 5C**) correlated significantly with IgG against both ends, particularly the C-terminal end (**Figure 5I**). Furthermore, the levels of anti-EBNA1_380-641_ but not anti-EBNA1_1-120_ IgG were significantly elevated in MS patients compared to HDs (**Figure 5J**).

Our *in vitro* data thus paralleled prior serological evidence demonstrating a selective increase in serum antibody levels against the C-terminal region of EBNA1 in MS patients (27, 34, 41, 42). Similarly, we observed a disease-associated increase of three MS autoantibodies for which cross-reactivity with EBNA1 had been demonstrated previously.

### 3.5 Antibody levels against the C-terminal portion of EBNA1 significantly correlate with autoantibodies targeting ANO2_1-275_ and GlialCAM_262-416_

**Figure 6:**
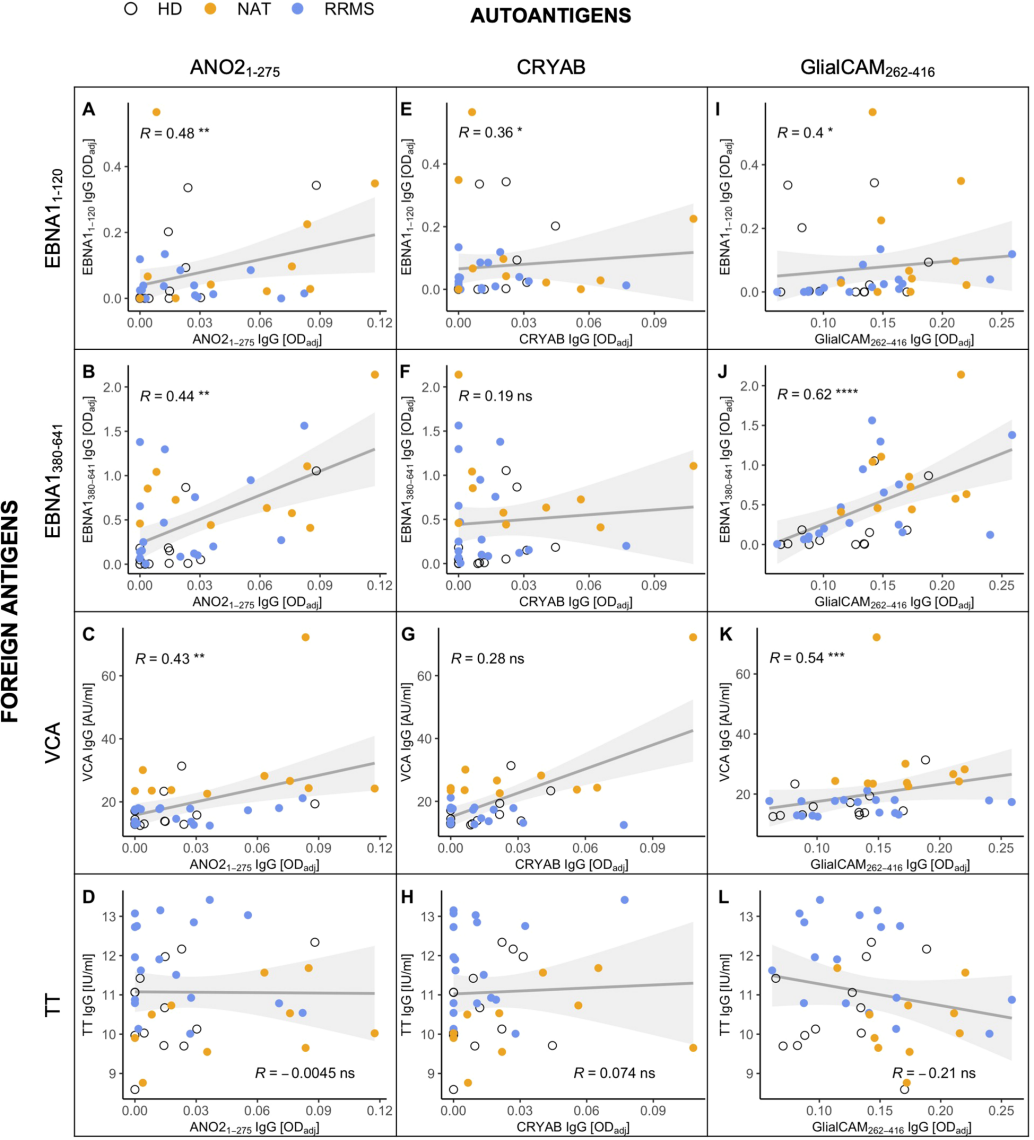
Correlations between the antibody levels against MS-associated autoantigens (x-axes) and foreign antigens (y-axes) in cell culture supernatants after stimulation *in vitro*. Foreign antigens included **(A, E, I)** the N-terminal (aa1-120) and **(B, F, J)** C-terminal (aa380-641) end of the EBNA1 protein, **(C, G, K)** the EBV viral capsid antigen (VCA), and **(D, H, L)** the common vaccine agent tetanus toxoid (TT), which served as an EBV-independent control. Autoantigens were selected due to previous studies demonstrating their antibody cross-reactivity with EBNA1. p = Spearman’s rank correlation. * p ≤ 0.05,** p ≤ 0.01, *** p ≤ 0.001, and **** p ≤ 0.0001. P-values above 0.05 were considered non-significant (ns).

Based on the increased levels of anti-EBNA1_380-641_ IgG in MS patients, we next investigated whether we could substantiate previous findings on antibody cross-reactivity between this region and the selected autoantigens *in vitro*. As a first step, we generated a matrix comparing the levels of autoantibodies against the antibodies for the foreign antigens. Interestingly, the antibody quantities against all three EBV-associated epitopes – EBNA1_1-120_, EBNA1_380-641_, and VCA – showed a significant positive correlation with the secretion of anti-ANO2_1-275_ (**Figures 6A–C**) and GlialCAM_262-416_ IgG (**Figures 6I–K**). Anti-CRYAB levels correlated significantly with EBNA1_1-120_ (**Figure 6E**), but not with the other EBV targets. None of the autoantibodies correlated with anti-TT IgG (**Figures 6D, H, L**), indicating that the B cell/antibody response against MS autoantigens and EBV was specific for this agent.

### 3.6 EBNA1_380-641_ can block the binding of anti-ANO2_1-275_ antibodies

**Figure 7:**
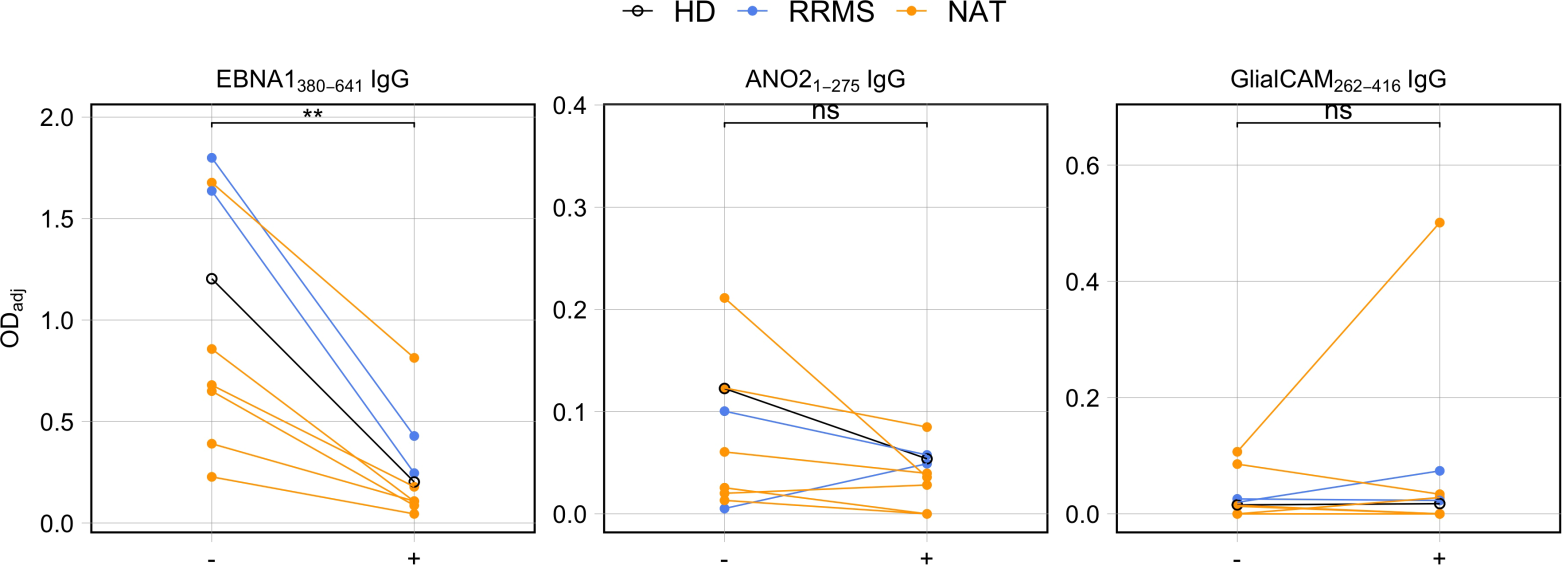
Antibody competition assays between the C-terminal end of EBNA1 (aa380-641) and the MS-autoantigens ANO2_1-275_ and GlialCAM_262-416_. Cell culture supernatants from stimulated PBMCs were incubated with EBNA1 aa380-641 protein (+) or PBST (–) overnight and subsequently tested for presence of antigen-specific IgG using ELISAs (p = Wilcoxon signed rank tests, HD: n = 1, RRMS: n = 2, NAT: n = 6). ** p ≤ 0.01. P-values above 0.05 were considered non-significant (ns).

Following up on the marked correlation between the C-terminal end of EBNA1 with both GlialCAM_262-416_ and ANO2_1-275_, we next assessed whether these antibodies indeed cross-recognized both antigens or whether the data mainly reflected that different antibody specificities are present at similar levels. Definitive proof of cross-recognition would require isolating and testing monoclonal antibodies individually. Since such experiments were beyond the scope of the present study, we performed a series of antibody cross-absorption assays on a subset of samples (n = 8) (**Figure 7**). In short, EBNA1_380-641_ protein was spiked at 30μM into supernatants obtained from stimulated cell culture supernatants and incubated overnight. By doing so, potentially cross-reactive antibodies should be absorbed by the protein, rendering them unable to bind to the cross-reactive autoantigen (ANO2_1-275_ or GlialCAM_262-416_) during immunosorbent assays the next day. As a negative control, PBST (PBS + 0.05% Tween™ 20) was added instead of EBNA1_380-641_. To confirm the efficacy of the absorption, we measured the levels of anti-EBNA1_380-641_ IgG in parallel to autoantibodies (**Figure 7A**). The results confirmed that spiking EBNA_380-641_ into the supernatants blocked the detection of anti-EBNA_380-641_ IgG. In line with the hypothesis of antibody cross-recognition, EBNA1_380-641_ induced a consistent reduction in antibody binding of anti-ANO2_1-275_ IgG, provided the unblocked reference signal (OD_adj_) was at least 0.05. Below this threshold, no blocking was observed, which contributed to the lack of significance overall (p = 0.0977). For GlialCAM_262-416_, all control conditions scored very low, and no consistent reduction in the binding to GlialCAM_262-416_ upon addition of EBNA1_380-641_ was observed. Altogether, our blocking experiments indicated that the C-terminal fragment of EBNA1 can interfere with the binding of ANO2_1-275_ antibodies, although this effect relied on the presence of adequate titers of the targeted antibodies.

### 3.7 The humoral EBNA1 antibody reactivity decreases after aHSCT

**Figure 8:**
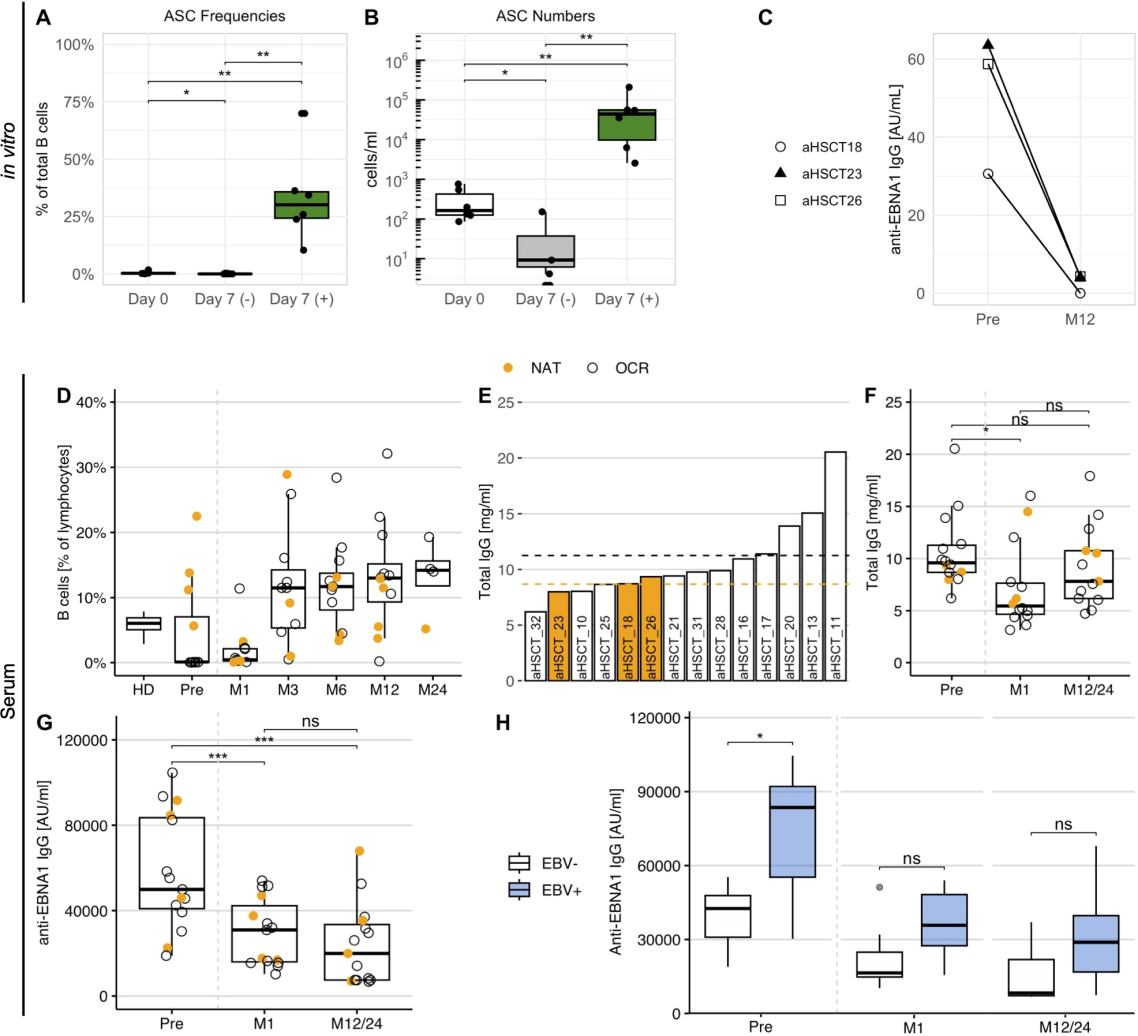
The anti-EBNA1 B cell reactivity in MS patients before and after aHSCT. **(A)** The percentage and **(B)** concentration of B cell ASCs in stimulated (+) and non-stimulated (–) cell culture supernatants of three aHSCT patients with previous NAT treatment. PBMCs for the expansion were obtained one month before as well as one year after aHSCT (p = Wilcoxon signed-rank tests with Holm’s correction). **(C)** The concentration of anti-EBNA1 IgG in stimulated cell culture supernatants before (Pre) and one year after aHSCT (M12) in aHSCT patients with prior NAT treatment. **(D)** The frequency of CD19^+^ B cells in untreated PBMC samples obtained one month before and at several time points after aHSCT. As a reference, average frequencies from HD1-16 were also included. **(E)** Total IgG in serum of aHSCT patients before transplantation, ordered by magnitude. Dotted lines represent the mean IgG for patients with NAT (orange) and OCR treatment (black). **(F)** Total IgG and **(G)** anti-EBNA1 IgG concentrations in serum before and after aHSCT (p = Wilcoxon signed-rank tests with Holm’s correction). **(H)** Anti-EBNA1 IgG in serum of patients with EBV reactivation (EBV+, n = 8) and without EBV reactivation (EBV–, n = 7) after aHSCT (p = Wilcoxon rank-sum tests with Holm’s correction). M, Month after aHSCT. * p ≤ 0.05,** p ≤ 0.01, *** p ≤ 0.001. P-values above 0.05 were considered non-significant (ns).

The above data indicated that the B cell response against EBNA1 is elevated in NAT-treated MS patients and that antibodies directed against the C-terminal portion of this protein also react with the autoantigen ANO2. We had previously shown that NAT treatment is associated with increased numbers of memory B cells (40), including those participating in a process called “autoproliferation”, a process that appears to be involved in the activation and expansion of autoreactive CD4^+^ cells (11). Based on these observations, we examined if a treatment that eliminates both T- and B cells and re-establishes new T- and B cell repertoires, i.e. aHSCT (18), would impact the increased anti-EBNA1 antibodies in NAT patients. For this purpose, we included three MS patients who had been treated with NAT and subsequently underwent aHSCT (aHSCT18, -23, -26) (**Supplementary Table 1**). For these individuals, we repeated the *in vitro* memory B cell expansion as in the previous samples, both for PBMCs obtained prior to aHSCT as well as 12 months thereafter. After confirming the expansion of ASCs within the cell cultures (**Figures 8A, B**), we quantified the EBNA1 IgG levels in both stimulated and non-stimulated cell cultures. Interestingly, stimulated B cells from all three patients secreted clearly fewer anti-EBNA1 antibodies one year after aHSCT (**Figure 8C**).

To assess whether similar changes could be observed in untreated patient serum, we tested sera from a total of 15 aHSCT patients, including the three patients above. Since 11 of these individuals had been treated with the B-cell depleting therapy ocrelizumab (OCR) before transplantation, we first quantified CD19^+^ B cells (**Figure 8D**) and total serum IgG before and after aHSCT (**Figures 8E, F**) to ensure that IgG titers were high enough to allow for an analysis of the humoral response. While B cells were efficiently depleted after aHSCT, their levels recovered within three months after transplantation (**Figure 8D**). IgG levels did not differ markedly between NAT and OCR patients before transplantation (**Figure 8E**), probably due to the relatively short treatment periods of less than 5 years. After aHSCT, IgG levels dropped slightly but differed no longer significantly from pre-treatment levels after 1 or 2 years, respectively (**Figure 8F**).

Different from total IgG, we observed a marked reduction in anti-EBNA1 IgG at one month after aHSCT, with a further decrease up to 1 to 2 years, respectively (**Figure 8G**). These observations supported that the *in vitro* antibody response mirrored changes in the serological EBNA1 reactivity in aHSCT patients. On top of this, our data indicated that the immunological effects of aHSCT led to a long-term decline in the humoral anti-EBNA1 response in these MS patients, eventually reflecting the reconstitution of a novel and distinct B cell repertoire.

### 3.8 Elevated serum anti-EBNA1 IgG is associated with EBV reactivation post-aHSCT

The *in vitro* decrease of anti-EBNA1 IgG following aHSCT raised the question of whether this observation could be linked to clinical observations relevant to aHSCT. Among common aHSCT-associated side effects, the reactivation of latent/persistent infections – including EBV – is among the common post-transplant adverse events (18). Viral reactivation can not only impact a progression-free survival (43) but also bears the risk of posttransplant lymphoproliferative disease (44). Considering the observed decline in anti-EBNA1 IgG titers after aHSCT, we compared these levels in patients experiencing EBV reactivation after transplantation (EBV+, n = 8) with those who did not (EBV–, n = 7). EBV reactivation was confirmed in aHSCT patients by quantitative PCR (≥122 EBV DNA copies/ml in blood). We found that patients experiencing an EBV reactivation after transplantation presented with significantly increased anti-EBNA1 IgG titers before aHSCT compared to those who did not (**Figure 8H**). Interestingly, this difference became even more pronounced after normalizing the anti-EBNA1 IgG to total serum IgG (**Supplementary Figure 7**). The distinction between patients with and without reactivation was no longer significant after transplantation but remained apparent for up to 2 years (**Figure 8H**). Together, these analyses pointed at an association between the strength of the anti-EBNA1 B cell response and the risk for EBV reactivation after aHSCT.

## 4 Discussion

In this study, we focus on the phenotypic and functional characterization of memory B cells in MS, which play a key role in this disease (45). Several B cell functions may be important in MS pathogenesis, including the secretion of pro-inflammatory cytokines (20, 21), the MHC class II-dependent presentation of autoantigen(s) to autoreactive CD4^+^ T cells (11, 24, 25), or immunoglobulin secretion (46, 47). Furthermore, memory B cells are the main reservoir during latent infection with EBV, the most important environmental risk factor for MS (2, 7). Antibodies against the EBV antigen EBNA1 (42, 48–50), which is one of the few EBV antigens expressed during latency (26), as well as prior infectious mononucleosis (2) substantially increase MS risk. This risk is further amplified in the context of the HLA-DR15 haplotype, hinting at a synergistic interaction of both factors (2, 36, 48, 51, 52). Among other mechanisms, mounting evidence from both B and T cells supports the idea that EBV contributes to the pathogenesis of MS through molecular mimicry with autoantigens (29, 32–34). Based on these findings, we investigated the memory B cell repertoire in MS patients and healthy donors with a focus on B cell immunoglobulin secretion, reactivity against EBNA1, and potential cross-reactivity of EBNA1 antibodies with MS autoantigens. For this purpose, we adopted an *in vitro* assay developed by Pinna et al. using a combination of IL-2 and the TLR7/8 agonist R848, which selectively activates and expands CD27^+^ memory B cells within total PBMCs and drives their differentiation into memory-derived ASCs (35). Our results demonstrated that this *in vitro* system was suitable to replicate previous serological data indicating both quantitative and qualitative (antigen-specific) changes in the secretion of EBNA1 antibodies in MS. Specifically, We validated earlier findings of increased IgG secretion against the C-terminal portion of EBNA1 in MS patients (27, 34, 41, 42) *in vitro* and confirmed that these correlated well with the serum titers of anti-EBNA1 in HDs and NAT patients. Interestingly, no such correlation was observed in the case of untreated RRMS patients, where quantities of EBNA1 IgG in supernatants were disproportionally lower compared to sera. A potential reason for this lack of congruency might consist of an ongoing or recent migration of EBV-specific B cells to the CNS in the context of MS pathogenesis (53). While lymphocyte migration across the BBB is prevented under NAT treatment (15), our hypothesis would explain why there were few EBNA1-reactive B cells in PBMC samples from untreated patients that could have been expanded *in vitro*. Contrary to this, anti-EBNA1 antibodies that had been secreted before migration may have endured in the periphery, which would account for the observed differences of anti-EBNA1 IgG *in vitro* and in serum.

We further showed that levels of autoantibodies against the MS autoantigens ANO2, CRYAB, and GlialCAM were elevated in NAT-treated MS patients *in vitro*. However, autoantibody titers were overall low in stimulated cell culture supernatants. This pointed to the technical difficulty related to the quantification of low-affinity and/or low-abundance antibodies, including those against self-antigens. In the future, more sensitive detection techniques such as cell-based assays may be superior to ELISAs as employed in this study (54). In addition, it may be advantageous to focus on the reactivity against distinct fractions of the protein sequences instead of using recombinant proteins. In our study, we implement this for GlialCAM (aa262-416) and ANO2 (aa1-275), but not for CRYAB. While it is certainly possible that the titers of anti-CRYAB antibodies were truly low in the samples, previous studies found that autoantibodies primarily recognize linear epitopes located at the N-terminal end (aa2-21) of the antigen (34). Hence, prior knowledge of the antibody-binding region within a target protein may be helpful to facilitate their detection in the future.

Beyond MS-associated differences in antigen-specific antibody levels *in vitro*, we observed a strong positive correlation between the IgG reactivity against the C-terminal region of EBNA1 and the two MS autoantigens ANO2_1-275_ and GlialCAM_262-428_. Our subsequent antibody blocking studies supported previous reports on cross-reactivity between EBNA1_380-641_ and ANO2_1-275_ (32). However, we note that efficient blocking depended on sufficient levels of target antibodies in the supernatants. These were consistently low in the case of anti-GlialCAM_262-416_, which may explain the lack of blocking upon addition of EBNA1_380-641_. To improve the competition efficacy, one could further enrich antibody titers in the samples by prolonging the memory B cell expansion *in vitro*. However, the lack of blocking between EBNA1 and GlialCAM could also be explained by the fact that the binding efficiency of anti-GlialCAM_262-416_ antibodies is influenced by post-translational modifications. In fact, the first study addressing antibody cross-reactivity between GlialCAM and EBNA1 highlighted that phosphorylation of the serine residue in position 376 of GlialCAM improved antibody binding by 50-fold (33). It is therefore also important to consider the effect of post-translational modifications on antibody binding.

In the last part of this study, we found that two highly effective treatments for MS – NAT and aHSCT – result in characteristic changes in the secretion patterns of anti-EBNA1 antibodies *in vitro*. In the case of NAT, the major mechanism of action consists of blocking the extravasation of peripheral leukocytes into the CNS (55), eventually trapping and potentiating pathogenic processes in the periphery (56, 57). Furthermore, NAT is associated with the release of memory B cells from secondary lymphoid organs and strong HLA-DR expression on B cells (40). These findings, together with the frequent rebound of disease activity after discontinuation of NAT (57), suggest that NAT promotes the expansion and activation of potentially autoreactive memory B cells and CD4^+^ T cells in the periphery (11, 40). In contrast, aHSCT leads to a complete and non-specific abrogation of the immune repertoire, followed by its subsequent reconstitution (18). Hence, the two therapies have very different and opposing effects on the peripheral immune system although both effectively limit the autoimmune reactions in the CNS. Our data demonstrate distinct effects of the two treatments on the peripheral EBNA1-directed memory B cell response, and that these observations are tied to their unique mechanism of action described above. While under NAT treatment, the humoral reactivity against EBNA1 appears to be potentiated, it is reduced after aHSCT. At last, we found that high serum anti-EBNA1 IgG was associated with an increased risk for EBV reactivation after transplantation. These data support that the heightened humoral response against EBNA1 in MS may not reflect an improved but rather an inefficient control of the virus (51).

Despite our focus on antibody secretion as a readout for B cell reactivity, it is worth mentioning that current data indicate that cross-reactive antibodies probably do not play a major pathogenic role in the disease in most MS patients (58). This is supported by the fact that highly effective B-cell-depleting therapies primarily target CD20^+^ B cells, but not antibody-secreting plasma cells (58). In fact, a therapy that primarily targeted antibody secretion via inhibition of the cytokines a proliferation-inducing ligand (APRIL) and B lymphocyte stimulator (BLyS), was associated with worsening of the disease (59). Hence, it appears that disease-specific immunoglobulin secretion patterns reflect changes in the B cell repertoire of MS patients, but that the pathologic consequences of these changes are manifested through other B cell functions. Recent evidence suggests that the key functions of B cells in MS may be antigen presentation to CD4^+^ T cells and secretion of proinflammatory cytokines (20, 21). Important findings in this context include that memory B cells and expression of HLA-DR are required for the activation and expansion of autoreactive brain-homing CD4^+^ T cells (11). Second, several studies have shown that CD4^+^ T cells recognize EBV-infected and/or EBV antigen-presenting B cells (60, 61). At last, the synergy between the key risk factors HLA-DR15 and EBV (2, 27, 51) indicates that the interaction between B and T cells via antigen presentation presents a crucial step in the pathogenesis. For the future, it will therefore be meaningful to extend our *in vitro* findings on memory B cells by considering the joint role of B and T cells in the context of EBV and to discern how molecular mimicry and cross-reactivity are mechanistically linked with the pathogenesis of MS.

## Data Availability

The original contributions presented in the study are included in the article/**Supplementary Material**, further inquiries can be directed to the corresponding author/s.
