# Supplementary material for: Enhanced and cross-reactive in vitro memory B cell response against Epstein-Barr virus nuclear antigen 1 in multiple sclerosis
Source: Front Immunol. 2024 Aug 27;15:1334720. doi: 10.3389/fimmu.2024.1334720 (PMC11385009; doi:10.3389/fimmu.2024.1334720)
Supplement: Supplementary file 1 [file Presentation1.pptx]

## Slide 1
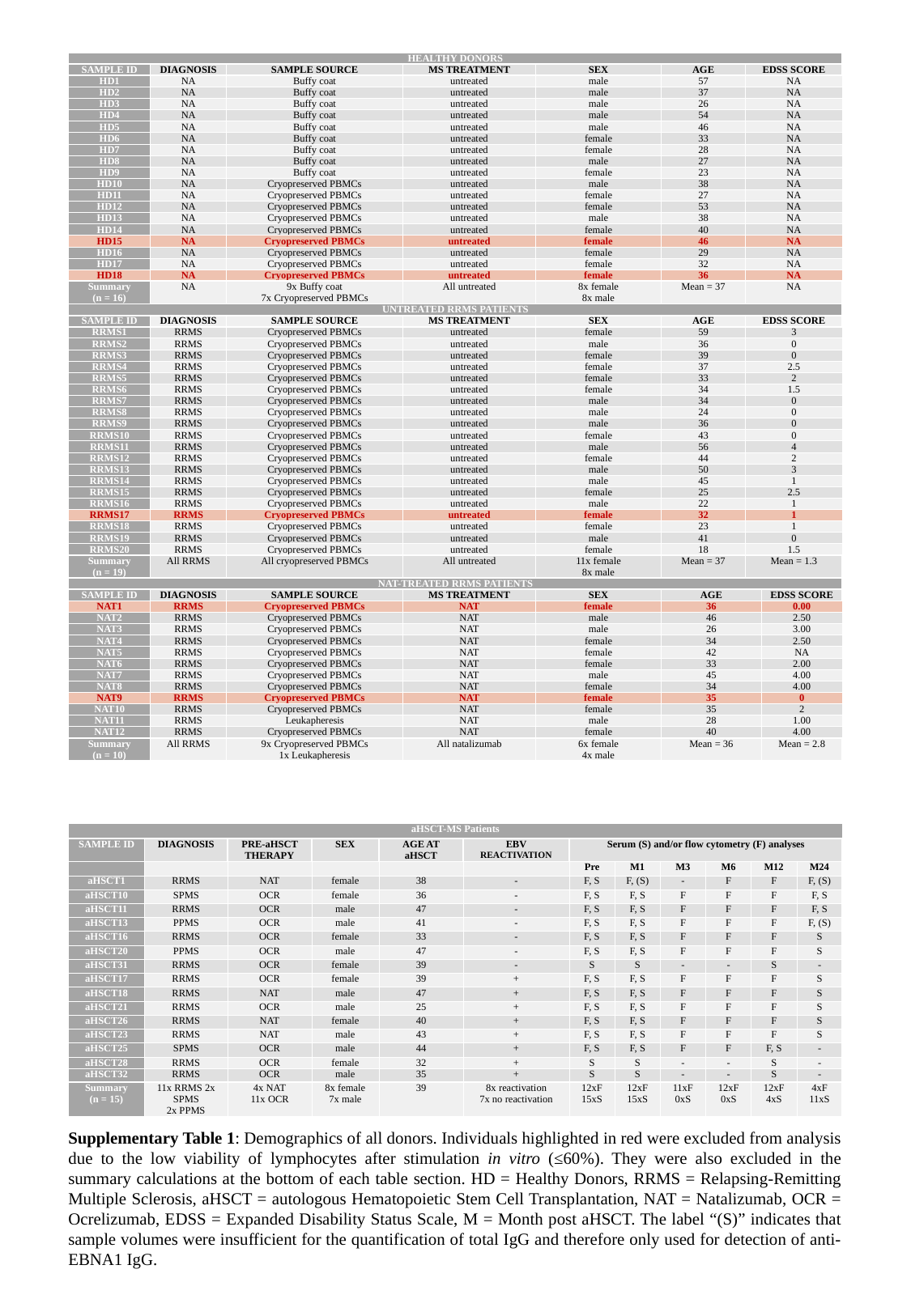

| HEALTHY DONORS | | | | | | | |
| --- | --- | --- | --- | --- | --- | --- | --- |
| SAMPLE ID | DIAGNOSIS | SAMPLE SOURCE | MS TREATMENT | SEX | AGE | EDSS SCORE | EDSS SCORE |
| HD1 | NA | Buffy coat | untreated | male | 57 | NA | NA |
| HD2 | NA | Buffy coat | untreated | male | 37 | NA | NA |
| HD3 | NA | Buffy coat | untreated | male | 26 | NA | NA |
| HD4 | NA | Buffy coat | untreated | male | 54 | NA | NA |
| HD5 | NA | Buffy coat | untreated | male | 46 | NA | NA |
| HD6 | NA | Buffy coat | untreated | female | 33 | NA | NA |
| HD7 | NA | Buffy coat | untreated | female | 28 | NA | NA |
| HD8 | NA | Buffy coat | untreated | male | 27 | NA | NA |
| HD9 | NA | Buffy coat | untreated | female | 23 | NA | NA |
| HD10 | NA | Cryopreserved PBMCs | untreated | male | 38 | NA | NA |
| HD11 | NA | Cryopreserved PBMCs | untreated | female | 27 | NA | NA |
| HD12 | NA | Cryopreserved PBMCs | untreated | female | 53 | NA | NA |
| HD13 | NA | Cryopreserved PBMCs | untreated | male | 38 | NA | NA |
| HD14 | NA | Cryopreserved PBMCs | untreated | female | 40 | NA | NA |
| HD15 | NA | Cryopreserved PBMCs | untreated | female | 46 | NA | NA |
| HD16 | NA | Cryopreserved PBMCs | untreated | female | 29 | NA | NA |
| HD17 | NA | Cryopreserved PBMCs | untreated | female | 32 | NA | NA |
| HD18 | NA | Cryopreserved PBMCs | untreated | female | 36 | NA | NA |
| Summary (n = 16) | NA | 9x Buffy coat 7x Cryopreserved PBMCs | All untreated | 8x female 8x male | Mean = 37 | NA | NA |
| UNTREATED RRMS PATIENTS | | | | | | | |
| SAMPLE ID | DIAGNOSIS | SAMPLE SOURCE | MS TREATMENT | SEX | AGE | EDSS SCORE | EDSS SCORE |
| RRMS1 | RRMS | Cryopreserved PBMCs | untreated | female | 59 | 3 | 3 |
| RRMS2 | RRMS | Cryopreserved PBMCs | untreated | male | 36 | 0 | 0 |
| RRMS3 | RRMS | Cryopreserved PBMCs | untreated | female | 39 | 0 | 0 |
| RRMS4 | RRMS | Cryopreserved PBMCs | untreated | female | 37 | 2.5 | 2.5 |
| RRMS5 | RRMS | Cryopreserved PBMCs | untreated | female | 33 | 2 | 2 |
| RRMS6 | RRMS | Cryopreserved PBMCs | untreated | female | 34 | 1.5 | 1.5 |
| RRMS7 | RRMS | Cryopreserved PBMCs | untreated | male | 34 | 0 | 0 |
| RRMS8 | RRMS | Cryopreserved PBMCs | untreated | male | 24 | 0 | 0 |
| RRMS9 | RRMS | Cryopreserved PBMCs | untreated | male | 36 | 0 | 0 |
| RRMS10 | RRMS | Cryopreserved PBMCs | untreated | female | 43 | 0 | 0 |
| RRMS11 | RRMS | Cryopreserved PBMCs | untreated | male | 56 | 4 | 4 |
| RRMS12 | RRMS | Cryopreserved PBMCs | untreated | female | 44 | 2 | 2 |
| RRMS13 | RRMS | Cryopreserved PBMCs | untreated | male | 50 | 3 | 3 |
| RRMS14 | RRMS | Cryopreserved PBMCs | untreated | male | 45 | 1 | 1 |
| RRMS15 | RRMS | Cryopreserved PBMCs | untreated | female | 25 | 2.5 | 2.5 |
| RRMS16 | RRMS | Cryopreserved PBMCs | untreated | male | 22 | 1 | 1 |
| RRMS17 | RRMS | Cryopreserved PBMCs | untreated | female | 32 | 1 | 1 |
| RRMS18 | RRMS | Cryopreserved PBMCs | untreated | female | 23 | 1 | 1 |
| RRMS19 | RRMS | Cryopreserved PBMCs | untreated | male | 41 | 0 | 0 |
| RRMS20 | RRMS | Cryopreserved PBMCs | untreated | female | 18 | 1.5 | 1.5 |
| Summary (n = 19) | All RRMS | All cryopreserved PBMCs | All untreated | 11x female 8x male | Mean = 37 | Mean = 1.3 | Mean = 1.3 |
| NAT-TREATED RRMS PATIENTS | | | | | | | |
| SAMPLE ID | DIAGNOSIS | SAMPLE SOURCE | MS TREATMENT | SEX | AGE | | EDSS SCORE |
| NAT1 | RRMS | Cryopreserved PBMCs | NAT | female | 36 | | 0.00 |
| NAT2 | RRMS | Cryopreserved PBMCs | NAT | male | 46 | | 2.50 |
| NAT3 | RRMS | Cryopreserved PBMCs | NAT | male | 26 | | 3.00 |
| NAT4 | RRMS | Cryopreserved PBMCs | NAT | female | 34 | | 2.50 |
| NAT5 | RRMS | Cryopreserved PBMCs | NAT | female | 42 | | NA |
| NAT6 | RRMS | Cryopreserved PBMCs | NAT | female | 33 | | 2.00 |
| NAT7 | RRMS | Cryopreserved PBMCs | NAT | male | 45 | | 4.00 |
| NAT8 | RRMS | Cryopreserved PBMCs | NAT | female | 34 | | 4.00 |
| NAT9 | RRMS | Cryopreserved PBMCs | NAT | female | 35 | | 0 |
| NAT10 | RRMS | Cryopreserved PBMCs | NAT | female | 35 | | 2 |
| NAT11 | RRMS | Leukapheresis | NAT | male | 28 | | 1.00 |
| NAT12 | RRMS | Cryopreserved PBMCs | NAT | female | 40 | | 4.00 |
| Summary (n = 10) | All RRMS | 9x Cryopreserved PBMCs 1x Leukapheresis | All natalizumab | 6x female 4x male | Mean = 36 | | Mean = 2.8 |
| aHSCT-MS Patients | | | | | | | | | | | |
| --- | --- | --- | --- | --- | --- | --- | --- | --- | --- | --- | --- |
| SAMPLE ID | DIAGNOSIS | PRE-aHSCT THERAPY | SEX | AGE AT aHSCT | EBV REACTIVATION | Serum (S) and/or flow cytometry (F) analyses | | | | | |
| | | | | | | Pre | M1 | M3 | M6 | M12 | M24 |
| aHSCT1 | RRMS | NAT | female | 38 | - | F, S | F, (S) | - | F | F | F, (S) |
| aHSCT10 | SPMS | OCR | female | 36 | - | F, S | F, S | F | F | F | F, S |
| aHSCT11 | RRMS | OCR | male | 47 | - | F, S | F, S | F | F | F | F, S |
| aHSCT13 | PPMS | OCR | male | 41 | - | F, S | F, S | F | F | F | F, (S) |
| aHSCT16 | RRMS | OCR | female | 33 | - | F, S | F, S | F | F | F | S |
| aHSCT20 | PPMS | OCR | male | 47 | - | F, S | F, S | F | F | F | S |
| aHSCT31 | RRMS | OCR | female | 39 | - | S | S | - | - | S | - |
| aHSCT17 | RRMS | OCR | female | 39 | + | F, S | F, S | F | F | F | S |
| aHSCT18 | RRMS | NAT | male | 47 | + | F, S | F, S | F | F | F | S |
| aHSCT21 | RRMS | OCR | male | 25 | + | F, S | F, S | F | F | F | S |
| aHSCT26 | RRMS | NAT | female | 40 | + | F, S | F, S | F | F | F | S |
| aHSCT23 | RRMS | NAT | male | 43 | + | F, S | F, S | F | F | F | S |
| aHSCT25 | SPMS | OCR | male | 44 | + | F, S | F, S | F | F | F, S | - |
| aHSCT28 | RRMS | OCR | female | 32 | + | S | S | - | - | S | - |
| aHSCT32 | RRMS | OCR | male | 35 | + | S | S | - | - | S | - |
| Summary (n = 15) | 11x RRMS 2x SPMS 2x PPMS | 4x NAT 11x OCR | 8x female 7x male | 39 | 8x reactivation 7x no reactivation | 12xF 15xS | 12xF 15xS | 11xF 0xS | 12xF 0xS | 12xF 4xS | 4xF 11xS |
Supplementary Table 1: Demographics of all donors. Individuals highlighted in red were excluded from analysis due to the low viability of lymphocytes after stimulation in vitro (≤60%). They were also excluded in the summary calculations at the bottom of each table section. HD = Healthy Donors, RRMS = Relapsing-Remitting Multiple Sclerosis, aHSCT = autologous Hematopoietic Stem Cell Transplantation, NAT = Natalizumab, OCR = Ocrelizumab, EDSS = Expanded Disability Status Scale, M = Month post aHSCT. The label “(S)” indicates that sample volumes were insufficient for the quantification of total IgG and therefore only used for detection of anti-EBNA1 IgG.

## Slide 2
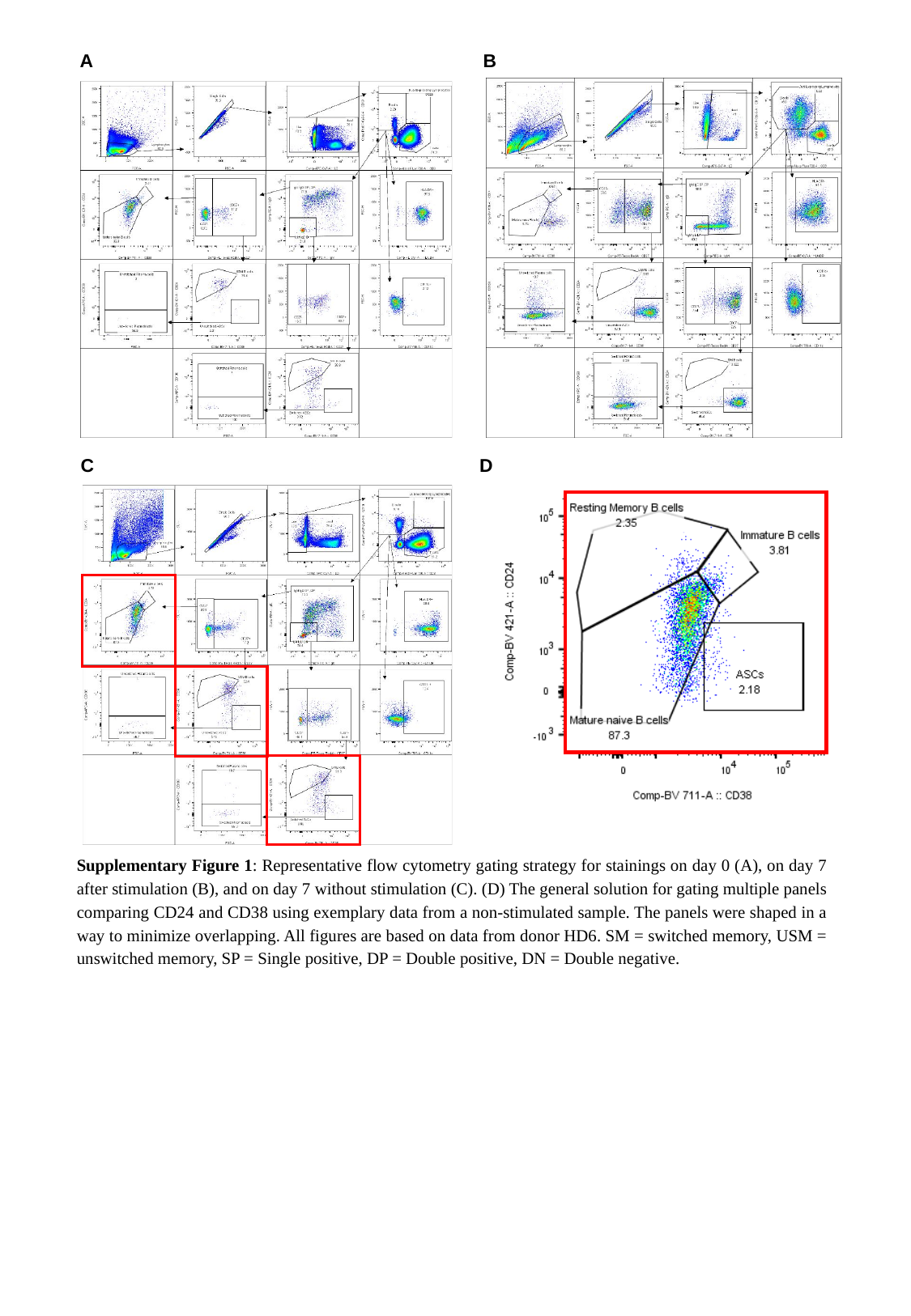

A
B
C
D
Supplementary Figure 1: Representative flow cytometry gating strategy for stainings on day 0 (A), on day 7 after stimulation (B), and on day 7 without stimulation (C). (D) The general solution for gating multiple panels comparing CD24 and CD38 using exemplary data from a non-stimulated sample. The panels were shaped in a way to minimize overlapping. All figures are based on data from donor HD6. SM = switched memory, USM = unswitched memory, SP = Single positive, DP = Double positive, DN = Double negative.

## Slide 3
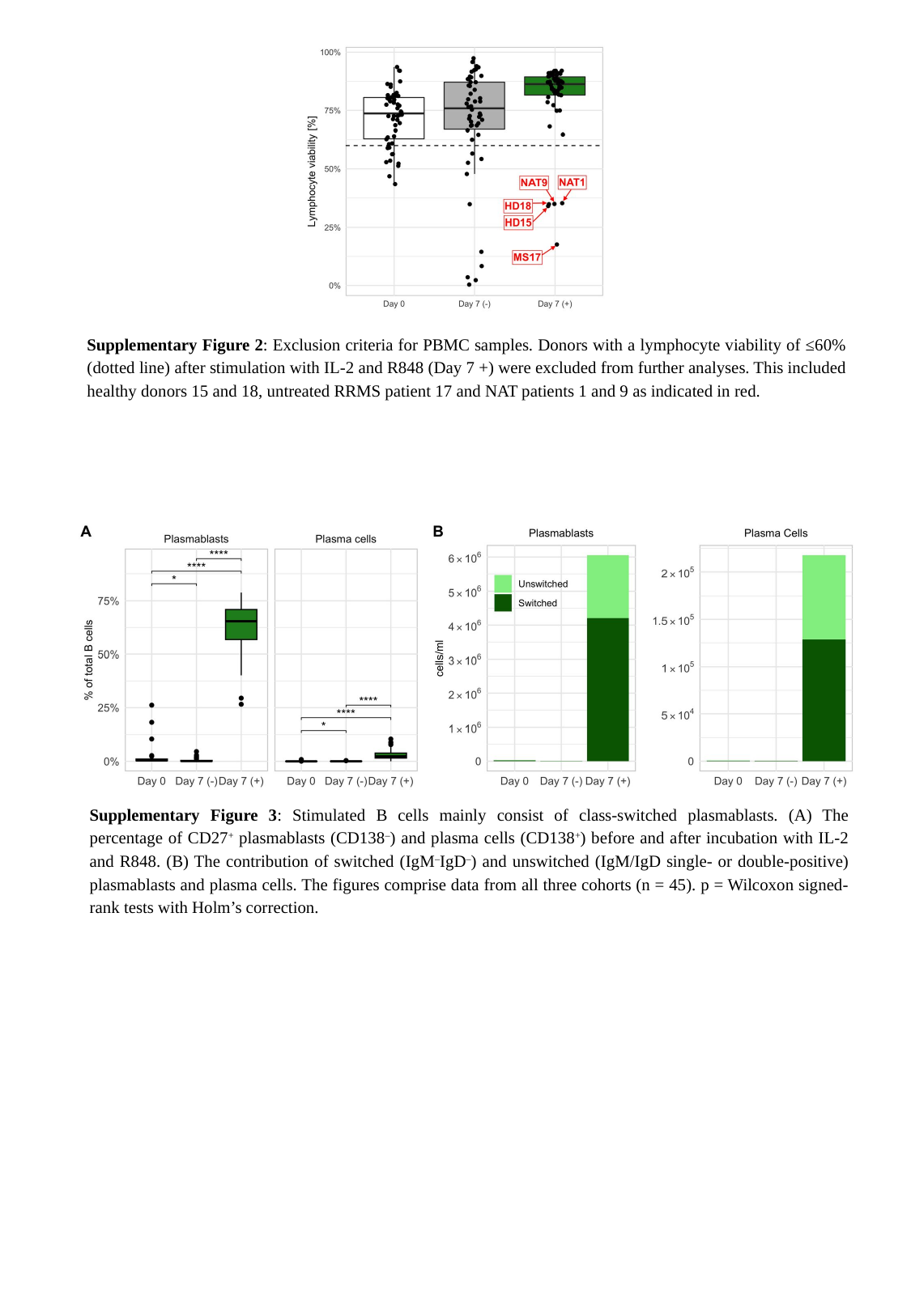

Supplementary Figure 2: Exclusion criteria for PBMC samples. Donors with a lymphocyte viability of ≤60% (dotted line) after stimulation with IL-2 and R848 (Day 7 +) were excluded from further analyses. This included healthy donors 15 and 18, untreated RRMS patient 17 and NAT patients 1 and 9 as indicated in red.
Supplementary Figure 3: Stimulated B cells mainly consist of class-switched plasmablasts. (A) The percentage of CD27+ plasmablasts (CD138–) and plasma cells (CD138+) before and after incubation with IL-2 and R848. (B) The contribution of switched (IgM–IgD–) and unswitched (IgM/IgD single- or double-positive) plasmablasts and plasma cells. The figures comprise data from all three cohorts (n = 45). p = Wilcoxon signed-rank tests with Holm’s correction.

## Slide 4
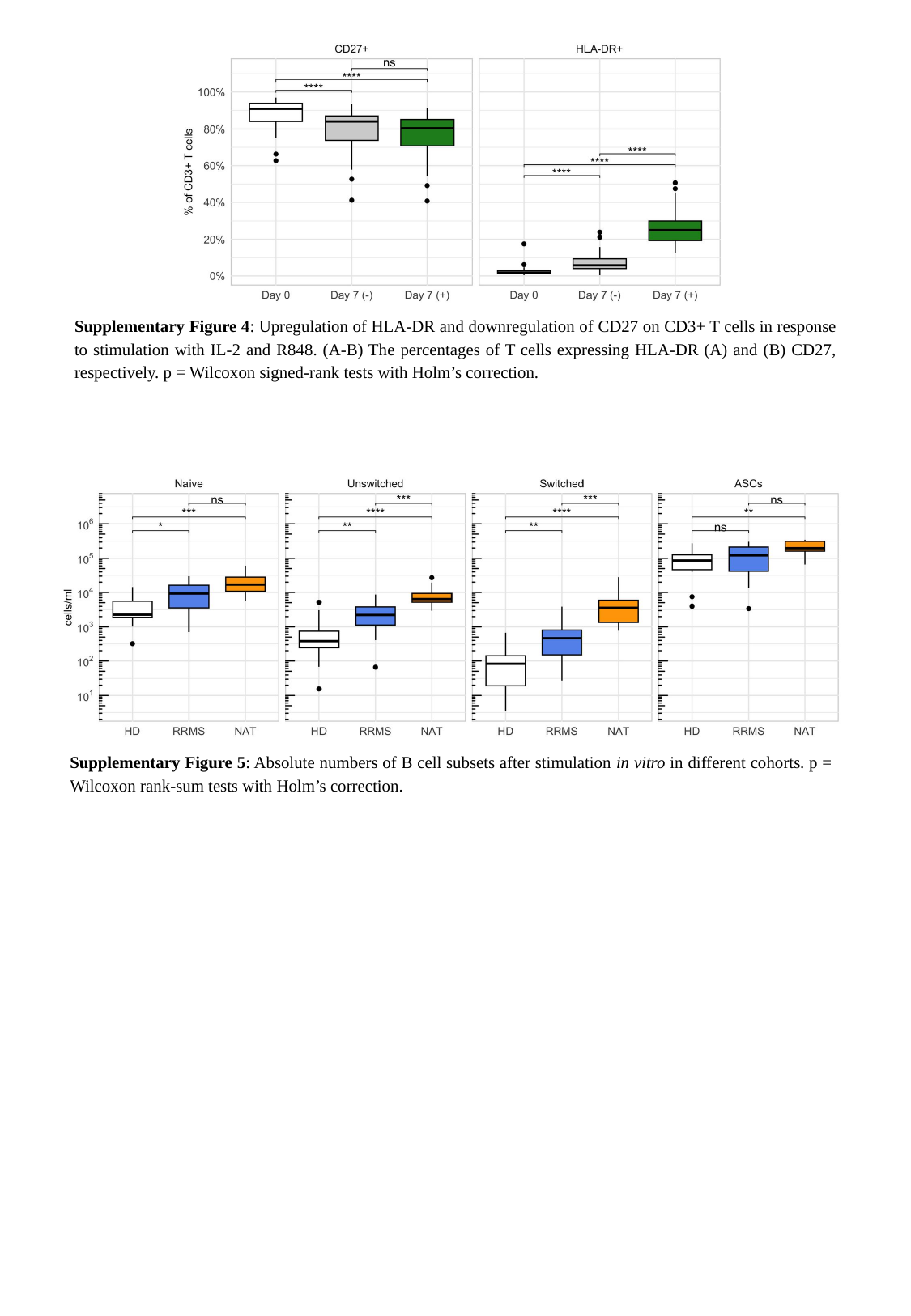

Supplementary Figure 4: Upregulation of HLA-DR and downregulation of CD27 on CD3+ T cells in response to stimulation with IL-2 and R848. (A-B) The percentages of T cells expressing HLA-DR (A) and (B) CD27, respectively. p = Wilcoxon signed-rank tests with Holm’s correction.
Supplementary Figure 5: Absolute numbers of B cell subsets after stimulation in vitro in different cohorts. p = Wilcoxon rank-sum tests with Holm’s correction.

## Slide 5
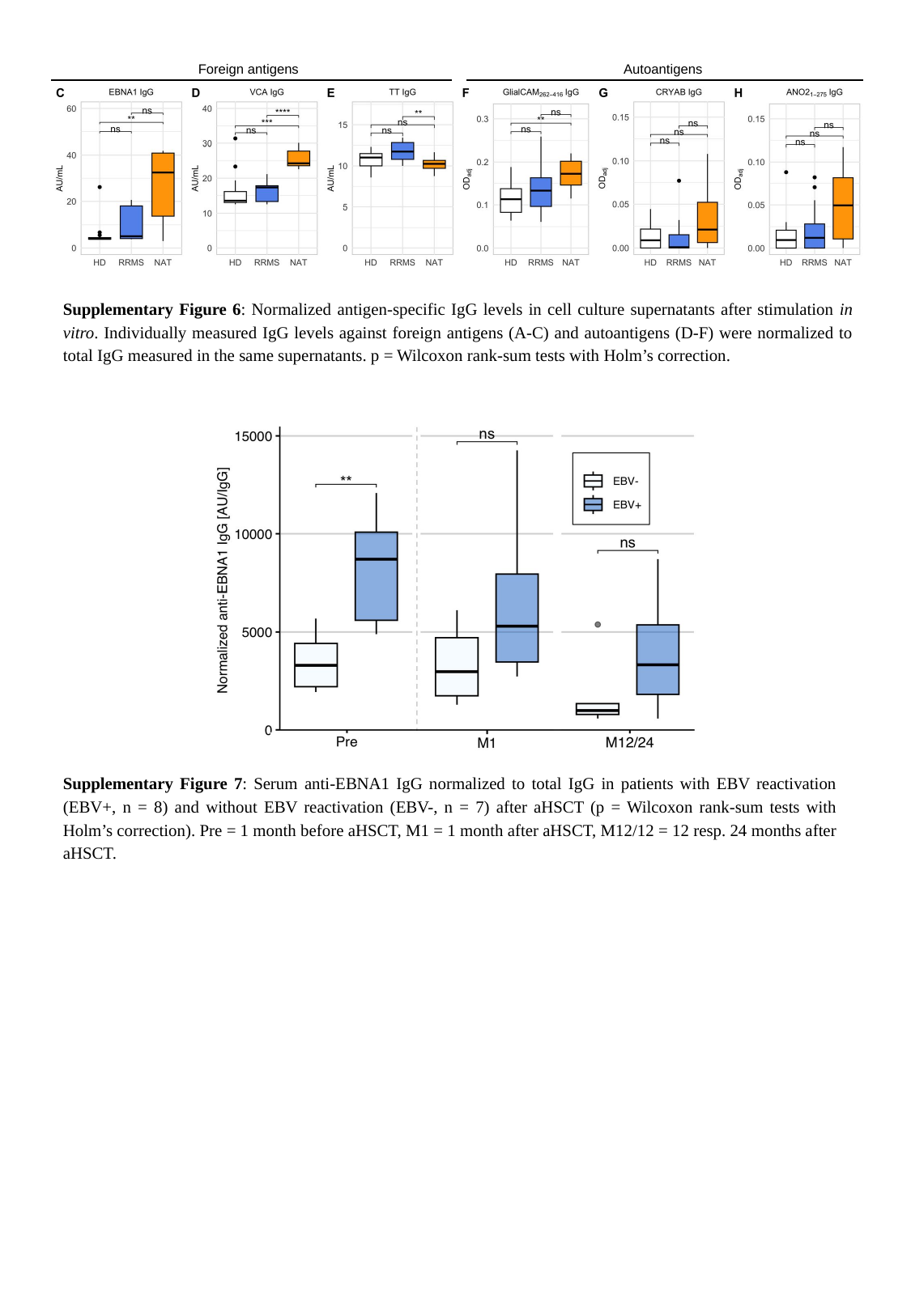

Foreign antigens
Autoantigens
Supplementary Figure 6: Normalized antigen-specific IgG levels in cell culture supernatants after stimulation in vitro. Individually measured IgG levels against foreign antigens (A-C) and autoantigens (D-F) were normalized to total IgG measured in the same supernatants. p = Wilcoxon rank-sum tests with Holm’s correction.
Supplementary Figure 7: Serum anti-EBNA1 IgG normalized to total IgG in patients with EBV reactivation (EBV+, n = 8) and without EBV reactivation (EBV-, n = 7) after aHSCT (p = Wilcoxon rank-sum tests with Holm’s correction). Pre = 1 month before aHSCT, M1 = 1 month after aHSCT, M12/12 = 12 resp. 24 months after aHSCT.
